# Alexithymic But Not Autistic Traits Impair Prosocial Behavior

**DOI:** 10.1007/s10803-021-05154-x

**Published:** 2021-06-28

**Authors:** Alexander Lischke, Harald J. Freyberger, Hans J. Grabe, Anett Mau-Moeller, Rike Pahnke

**Affiliations:** 1 Department of Psychology, Medical School Hamburg, Am Kaiserkai 1, 20457 Hamburg, Germany; 2 Department of Psychology, University of Greifswald, Franz-Mehring-Str. 47, 17489 Greifswald, Germany; 3 Department of Psychiatry and Psychotherapy, University Medicine Greifswald, Ellernholzstr. 1-2, 17475 Greifswald, Germany; 4 Department of Sport Science, University of Rostock, Ulmenstr. 69, 18057 Rostock, Germany

**Keywords:** Social value orientation, Cooperation, Autism, Alexithymia, Empathy

## Abstract

Social impairments are a core feature of autism-spectrum disorders. However, there is a considerable variability in these impairments. Most autistic individuals show large impairments in social functioning but some autistic individuals show small impairments in social functioning. The variability of these impairments has been attributed to the presence or absence of alexithymia. To address this issue, we capitalized on the fact that alexithymic and autistic traits are broadly distributed in the population. This allowed us to investigate how alexithymic and autistic traits affect social functioning in healthy individuals. Healthy individuals showed impairments on a resource-allocation task that were due to alexithymic but not autistic traits. These findings suggest that alexithymic rather than autistic traits impair prosocial behavior across the autism-spectrum.

## Introduction

Autism-spectrum disorder (ASD) is a clinical condition that is characterized by impairments in social cognition and social interaction (APA, 2013). Although social impairments are common among autistic individuals (Velikonja et al., 2019), there is a considerable variability in these impairments. The processing of others’ emotions, for instance, varies considerably among autistic individuals (Harmset al., 2010). Most autistic individuals show large impairments in emotion processing but some autistic individuals show small impairments in emotion processing. The variability of these and other impairments may depend on the presence or absence of alexithymia (Bird & Cook, 2013). Alexithymia is a non-clinical condition that is characterized by difficulties in identifying and describing one’s own emotions (Nemiah et al., 1976). Considering that emotions serve as guidance in many social contexts (Keltner & Haidt, 1999), it is not surprising that alexithymia is often associated with impairments in social cognition and social interaction (Grynberg et al., 2018). Alexithymia is quite prevalent among autistic individuals (Kinnaird et al., 2019), implying that autistic individuals with high levels of alexithymia may be more impaired in social cognition and social interaction than autistic individuals with low levels of alexithymia. Autistic individuals with high levels of alexithymia show indeed more impairments in social cognition than autistic individuals with low levels of alexithymia. Emotion recognition or empathetic responding, for instance, is more impaired in autistic individuals with high than low levels of alexithymia (Bird et al., 2010; Cook et al., 2013; Silani et al., 2008). We, thus, assume that impairments in social interaction are also more pronounced among autistic individual with high than low levels of alexithymia.

To test this assumption, we capitalized on the fact that autistic and alexithymic traits are broadly distributed in the population (Franz et al., 2008; Ruzich et al., 2015). This allowed us to investigate how autistic and alexithymic traits impair social interaction in healthy individuals. Impairments in social interaction can be modelled with economic tasks that operationalize social interaction in terms of prosocial behavior (King-Casas & Chiu, 2012). Following this approach, we administered a resource allocation task to a sample of healthy individuals whose autistic and alexithymic traits had been determined with personality questionnaires. Similar as in previous investigations (Brewer et al., 2015; Cook et al., 2013), we performed correlation and regression analyses to investigate associations between task performance and personality traits. Assuming that task performance would be more impaired by alexithymic than by autistic traits (Bird & Cook, 2013), we expected alexithymic rather than autistic traits to be negatively associated with prosocial behavior on the resource allocation task.

## Method

### Participants

Seventy-four healthy individuals (ethnicity: Caucasian, age range: 18–35 years, educational level: higher education) participated in the study. None of the participants was or had been in psychotherapeutic or psychopharmacological treatment. A power analysis with G*Power (Faul et al., 2007) indicated that the number of participants was large enough to detect medium sized associations between prosocial behavior and autistic or alexithmic traits in the planned analyses (correlation analyses, one-sided, and regression analyses, two-sided: *α* = 0.05, 1 − *β* = 80, *r* = 0.30, *f*^*2*^ = 0.15). All participants provided written informed consent to the study protocol that was approved by the ethics committee of the University of Rostock and carried out in accordance with the Declaration of Helsinki.

### Questionnaires

We used in-house questionnaires for the assessment of participants’ demographical characteristics (age, sex, education) and established questionnaires for the assessment of participants’ psychological characteristics (psychopathology, autism, alexithymia). Psychopathological symptoms were assessed with the depression and anxiety scales of the Brief Symptom Inventory (BSI; Derogatis, 2000), autistic traits were assessed with the Autism Spectrum Quotient 10 (AQ-10; Allison et al., 2012; Baron-Cohen et al., 2001) and alexithymic traits were assessed with the Toronto Alexithymia Scale 20 (TAS-20; Bagby et al., 1994a, 1994b; Parker et al., 2003).

### Task

**Fig. 1:**
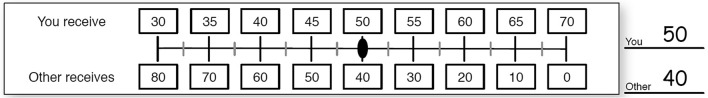
Example of a continuum of self-other payoff allocations that were used in the Social Value Orientation Task (SVO; Murphy et al., 2011)

We used the Social Value Orientation test (Murphy et al., 2011), a resource allocation task, to assess participants’ pro-social behavior via a computer interface (Lischke et al., 2018). The SVO comprised six items with a choice over a defined continuum of self-other payoff allocations (see Fig. 1). Participants had to select payoff allocations that reflected their most preferred payoffs for themselves and another participant whose identity remained anonymous throughout the study. On basis of these selections, the inverse ratio between the mean payoffs for the self and the other was calculated. The resulting index, the social value orientation angle (SVO-A), reflected participants’ preferences for pro-social allocations (i.e., allocations with higher payoffs for the other than for the self) as compared to anti-social allocations (i.e., allocations with lower payoffs for the other than for the self). Higher SVO-A values indicated that participants displayed prosocial behavior (upper limit: 61.39°) and lower SVO-A values indicated that participants displayed anti-social behavior (lower limit: − 16.26°).

### Statistical Analysis

We used SPSS 22 (SPSS Inc., Chicago, IL, USA) for all analyses. Our preliminary analyses of participants’ task performance revealed invalid allocation selections (i.e. allocations outside of the range of possible allocations). These invalid selections compromised the determination of the social value orientation index (Murphy et al., 2011), limiting the number of participants that could be considered in our main analyses (*n* = 67; see Table 1). Our main analyses comprised correlation and regression analyses, which were performed with bootstrapping (10,000 samples) to control for deviations from normality (Wright et al., 2011). Whereas the correlation analyses allowed us to explore associations between participants’ personality traits and participants’ task performance, the regression analyses allowed us to investigate associations between participants’ personality traits and participants’ task performance in more detail. To rule out that the results of the correlation and regression analyses were affected by other participant characteristics than participants’ personality traits (Hendryx et al., 1991; Kanai et al., 2011), we controlled for differences in participants’ age, sex, depression and anxiety in all analyses. We set the significance level for these analyses at *p* ≤ 0.05 (corrected for multiple comparisons) and determined significance values (*p*), effect size measures (*r*, *R*^*2*^, Δ*R*^*2*^, *B, z, q*) and 95% confidence intervals (CIs) to facilitate the interpretation of the corresponding results.

**Table 1:** Participant characteristics

	*M (SE M)/N*
Sex (m/f)	33/34
Age (years)	26.10 (0.50)
Anxiety (BSI-ANX)	0.53 (0.06)
Depression (BSI-DEP)	0.35 (0.05)
Alexithymia (TAS-20)	43.03 (1.22)
Autism (AQ-10)	2.19 (0.14)
Cooperation (SVO-A)	32.16 (1.29)

*m* male, *f* female, *BSI-ANX* Brief Symptom Inventory—Anxiety Scale (Derogatis, 2000), *BSI-DEP* Brief Symptom Inventory—Depression Scale (Derogatis, 2000), *TAS*-*20* Toronto Alexithymia Scale 20 (Bagby et al., 1994a, 1994b; Parker et al., 2003), *AQ-10* Autism Spectrum Quotient 10 (Allison et al., 2012; Baron-Cohen et al., 2001), *SVO-A* Social Value Orientation—Angle (Murphy et al., 2011)

## Results

**Fig. 2:**
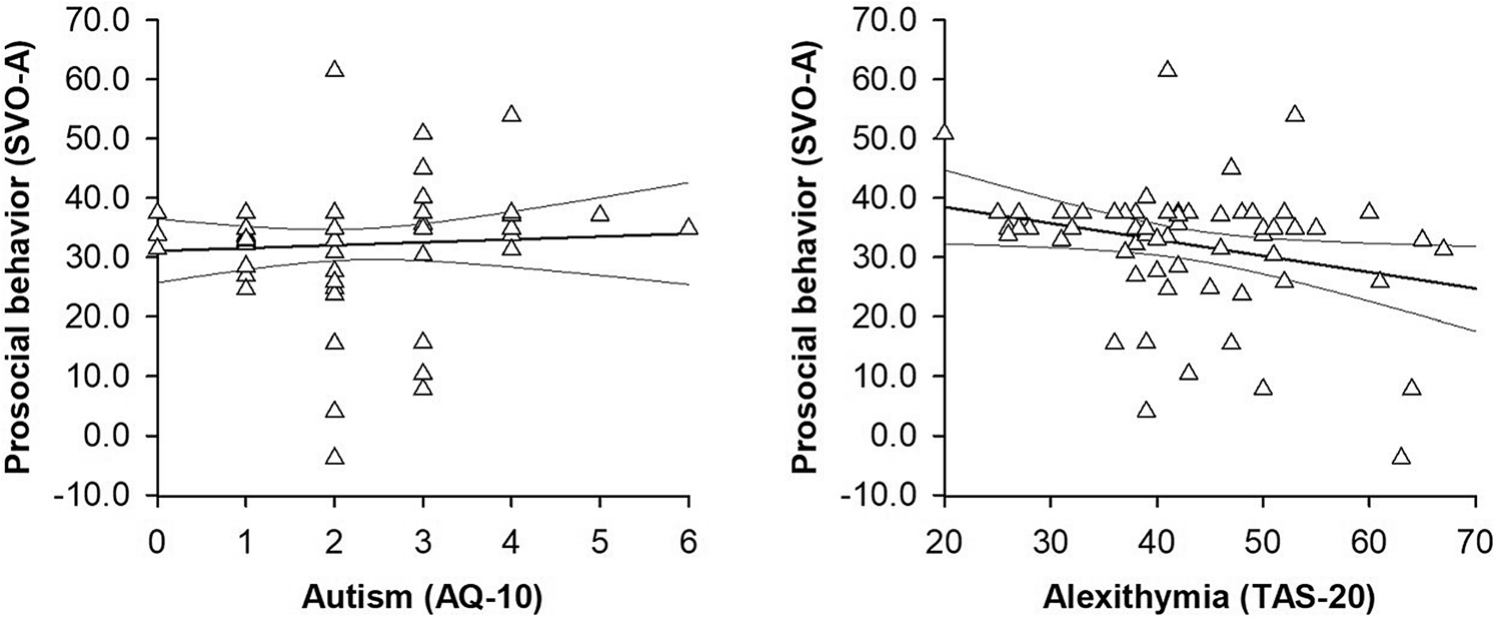
Scatterplots with lines of best fit and 95% confidence intervals demonstrating associations between participants’ prosocial behavior and participants’ (*left panel*) autistic or (*right panel*) alexithymic traits. Prosocial behavior was assessed with the Social Value Orientation Angle (SVO-A; Murphy et al., 2011), autism was assessed with the Autism Spectrum Questionnaire 10 (AQ-10; Allison et al., 2012; Baron-Cohen et al., 2001) and alexithymia was assessed with the Toronto Alexithymia Scale 20 (TAS-20; Bagby et al., 1994a, 1994b; Parker et al., 2003)

We run a series of correlation analyses to explore associations between participants’ personality traits and participants’ task performance. To control for participant characteristics that may affect these associations (age, sex, depression, anxiety), we performed partial instead of full correlations. We found a positive association between participants’ autistic and alexithymic traits (*r*(61) = 0.32, *p* = 0.011, 95% CI [0.06, 0.53]). Whereas participants’ autistic symptoms were not associated with participants’ prosocial behavior (*r*(61) = 0.07, *p* = 0.591, 95% CI [− 0.15, 0.24], see Fig. 2), participants’ alexithymic symptoms were negatively associated with participants’ prosocial behavior (*r*(61) = − 0.29, *p* = 0.022, 95% CI [− 0.50, − 0.04], see Fig. 1). A formal comparison of the correlation coefficients that were obtained in these analyses confirmed that participants’ autistic and alexithymic traits were differentially associated with participants’ prosocial behavior (*z* = 2.52, *p* = 0.006, *q* = 0.37).

We run a series of regression analyses to further investigate the associations between participants’ personality traits and participants’ task performance. To control for participant characteristics that may affect these associations (age, sex, depression, anxiety), we entered these participant characteristics before participants’ autistic and alexithymic traits into the respective regression models. Whereas participants’ autistic traits were entered before participants’ alexithymic traits into one regression model (model one), participants’ autistic traits were entered after participants’ alexithymic traits into another regression model (model two). Varying the order of participants’ alexithymic and autistic traits in the regression models allowed us to control the close association between these traits (Brewer et al., 2015; Cook et al., 2013). Regardless whether we entered participants’ autistic traits before or after participants’ alexithymic traits into the regression model (see Table 2), we found no association between participants’ autistic traits and participants’ prosocial behavior (model one (step 3): *B* = 1.55, 95% CI [− 0.46, 3.17], *t*(60) = 1.39, *p* = 0.090; model two (step 2): *B* = 0.60, 95% CI [− 1.45, 2.14], *t*(61) = 0.54, *p* = 0.461). As a consequence, participants’ autistic traits failed to account for a substantial proportion of participants’ prosocial behavior (model one (step 3): Δ*R*^2^ = 0.03, Δ*F*(1, 60) = 1.94, *p* = 0.169; model two (step 2): Δ*R*^2^ = 0.00, Δ*F*(1, 61) = 0.29, *p* = 0.591). We found, however, a negative association between participants’ alexithymic traits and participants’ prosocial behavior [model one (step 2): *B* = − 0.35, 95% CI [− 0.59, − 0.07], *t*(61) = − 2.35, *p* = 0.022; model two (step 3): *B* = − 0.42, 95% CI [− 0.68, − 0.10], *t*(60) = − 2.69, *p* = 0.009]. The association emerged regardless whether participants’ alexithymic traits were entered before or after participants’ autistic traits (see Table 2). Consequently, participants’ alexithymic traits accounted for a substantial proportion of participants’ prosocial behavior [model one (step 2): Δ*R*^2^ = 0.08, Δ*F*(1, 61) = 5.50, *p* = 0.022; model two (step 3): Δ*R*^2^ = 0.10, Δ*F*(1, 60) = 7.21, *p* = 0.009].

**Table 2:** Associations between participants’ prosocial behavior and participants’ autistic or alexithymic traits

Model one	Prosocial behavior (SVO-A)					Model two	Prosocial behavior (SVO-A)				
	*B*	*SE B*	95% CI	*t*	*p*		*B*	*SE B*	95% CI	*t*	*p*
*Step one*						*Step one*					
Sex	− 0.38	0.36	[− 1.03, 0.33]	− 1.15	0.293	Sex	− 0.38	0.37	[− 1.07, 0.38]	− 1.15	0.326
Age (years)	1.46	2.53	[− 3.53, 6.46]	0.55	0.565	Age (years)	1.46	2.68	[− 3.91, 6.64]	0.55	0.604
Anxiety (BSI-ANX)	− 2.25	3.17	[− 8.73, 3.70]	− 0.75	0.486	Anxiety (BSI-ANX)	− 2.25	3.10	[− 8.00, 3.86]	− 0.75	0.503
Depression (BSI-DEP)	− 0.94	5.57	[− 13.08, 8.53]	− 0.27	0.867	Depression (BSI-DEP)	− 0.94	5.64	[− 13.67, 8.25]	− 0.27	0.860
*Step two*						*Step two*					
Sex	− 0.46	0.36	[− 1.12, 0.27]	− 1.45	0.217	Sex	− 0.36	0.37	[− 1.06, 0.41]	− 1.11	0.331
Age (years)	3.27	2.76	[− 2.53, 8.53]	1.22	0.259	Age (years)	1.45	2.71	[− 3.91, 6.79]	0.54	0.593
Anxiety (BSI-ANX)	0.83	3.51	[− 6.35, 7.50]	0.26	0.808	Anxiety (BSI-ANX)	− 2.49	3.21	[− 8.26, 4.09]	− 0.81	0.463
Depression (BSI-DEP)	0.79	5.29	[− 10.84, 10.01]	0.23	0.856	Depression (BSI-DEP)	− 0.98	5.64	[− 13.92, 8.26]	− 0.28	0.860
Alexithymia (TAS-20)	− 0.35	0.14	[− 0.59, − 0.07]	− 2.35	0.022*	Autism (AQ-10)	0.60	0.87	[− 1.45, 2.14]	0.54	0.461
*Step three*						*Step three*					
Sex	− 0.45	0.34	[− 1.06, 0.26]	− 1.42	0.211	Sex	− 0.45	0.36	[− 1.11, 0.33]	− 1.42	0.212
Age (years)	3.60	2.83	[− 2.19, 9.27]	1.35	0.219	Age (years)	3.60	3.01	[− 2.71, 9.33]	1.35	0.257
Anxiety (BSI-ANX)	0.80	3.45	[− 6.49, 7.39]	0.25	0.800	Anxiety (BSI-ANX)	0.80	3.31	[− 5.70, 7.34]	0.25	0.797
Depression (BSI-DEP)	1.04	5.03	[− 10.25, 9.96]	0.30	0.811	Depression (BSI-DEP)	1.04	4.97	[− 10.24, 9.5]	0.30	0.833
Alexithymia (TAS-20)	− 0.42	0.15	[− 0.68, − 0.11]	− 2.69	0.011*	Autism (AQ-10)	1.55	0.95	[− 0.55, 3.34]	1.39	0.088
Autism (AQ-10)	1.55	0.92	[− 0.46, 3.17]	1.39	0.090	Alexithymia (TAS)	− 0.42	0.15	[− 0.68, − 0.10]	− 2.69	0.009**

Model one: step one: *R*^2^ = 0.04, *F*(4, 62) = 0.59, *p* = 0.670, step two: Δ*R*^2^ = 0.08, Δ*F*(1, 61) = 5.50, *p* = 0.022*, step three: Δ*R*^2^ = 0.03, Δ*F*(1, 60) = 1.94, *p* = 0.169; model two: step one: *R*^2^ = 0.04, *F*(4, 62) = 0.59, *p* = 0.670, Step 2: Δ*R*^2^ = 0.00, Δ*F*(1, 61) = 0.29, *p* = 0.591, step three: Δ*R*^2^ = 0.10, Δ*F*(1, 60) = 7.21, *p* =0 .009**; *SVO-A* Social Value Orientation—Angle (Murphy et al., 2011), *BSI-ANX* Brief Symptom Inventory—Anxiety Scale (Derogatis, 2000), *BSI-DEP* Brief Symptom Inventory—Depression Scale (Derogatis, 2000), *AQ-10* Autism Spectrum Quotient 10 (Allison et al., 2012; Baron-Cohen et al., 2001), *TAS-20* Toronto Alexithymia Scale 20 (Bagby et al., 1994a, 1994b; Parker et al., 2003)

*p ≤ 0.05, **p ≤0 .01

## Discussion

To test whether alexithymic rather than autistic traits account for impairments in prosocial behavior, we administered a resource allocation task to a sample of healthy individuals whose autistic and alexithymic traits had been determined with personality questionnaires. Our well-powered and well-controlled analyses revealed the expected pattern of associations between task performance and personality traits: Individuals’ alexithymic traits were negatively associated with individuals’ prosocial behavior, indicating that individuals with high levels of alexithymia displayed less prosocial behavior than individuals with low levels of alexithymia. Individuals’ autistic traits, on the contrary, were neither positively nor negatively associated with individuals’ prosocial behavior, indicating that individuals with high levels of autism displayed as much prosocial behavior as individuals with low levels of autism. We, thus, assume that alexithymic rather than autistic traits impair prosocial behavior. To validate this assumption, our investigation has to be replicated with individuals who show a larger variability in alexithymic and autistic traits than our individuals. Investigations with autistic individuals and their first-degree relatives may be particularly useful for this purpose (Berthoz et al., 2013; Szatmari et al., 2008).

To understand why alexithymic rather than autistic traits impair prosocial behavior, it may be helpful to consider how alexithymia affects empathy in healthy and autistic individuals. Empathy, the ability to share and understand the emotions or thoughts of others, is a powerful motivator of prosocial behavior (Decety et al., 2016). Healthy individuals with high levels of empathy show more prosocial behavior than healthy individuals with low levels of empathy (Edele et al., 2013; Jordan et al., 2016), implying that alterations in empathy lead to profound alterations in prosocial behavior. Alexithymia alters empathy in healthy individuals (Grynberg et al., 2018). Healthy individuals with high levels of alexithymia are less able to share and understand the feelings of others than healthy individuals with low levels of alexithymia (Moriguchi et al., 2006, 2007; Parker et al., 2001). However, alexithymia also alters empathy in autistic individuals (Grynberg et al., 2018). Autistic individuals with high levels of alexithymia are also less able to share and understand the feelings of others than autistic individuals with low levels of alexithymia (Bird et al., 2010; Mul et al., 2018; Silani et al., 2008). We, thus, assume that alexithymia impairs prosocial behavior in healthy and autistic individuals by altering empathetic abilities that are relevant for the display of prosocial behavior (Decety et al., 2016). Although these assumptions appear to be somewhat speculative, we would like to point out that it has already been shown that alexithymia-dependent alterations of empathetic processes impair prosocial behavior among healthy individuals (Feldmanhall et al.,^2013^). Considering that autistic individuals display much higher levels of alexithymia and much lower levels of empathy than healthy individuals (Berthoz et al., 2013), we believe that alexithymia-dependent alterations of empathetic processes also contribute to impairments in prosocial behavior among autistic individuals.

We investigated how alexithymic and autistic traits impair prosocial aspects of social interaction, whereas others investigated how alexithymic and autistic traits impair emotional aspects of social cognition (Bird et al., 2010; Cook et al., 2013; Oakley et al., 2016; Silani et al., 2008). Although these investigations focused on impairments in different social domains, they nonetheless help to explain why some but not all autistic individuals show impairments in emotion recognition (Adolphs et al., 2001; Humphreys et al., 2007; Otsuka et al., 2017), empathetic responding (Dziobek et al., 2008; Hadjikhani et al., 2014; Rogers et al., 2007) and prosocial acting (Cage et al., , 2013; Ikuse et al., 2018; Izuma et al., 2011). Autistic individuals with high levels of alexithymia are more likely to display these and other impairments than autistic individuals with low levels of alexithymia (Bird & Cook, 2013). Given that the absence or presence of alexithymia has such profound effects on social functioning, we think that it is time to reconsider the current practice of diagnosing and treating ASD (Bird & Cook, 2013; Hobson et al., 2020). We believe that a thorough assessment of alexithymic and autistic traits facilitates the identification of individuals who benefit more from alexithymia-specific than autism-specific treatment approaches. We, therefore, hope that our investigation opens an avenue for novel approaches to the diagnosis and treatment of autistic individuals with different alexithymia levels.
